# Effects of Maximal Sodium and Potassium Conductance on the Stability of Hodgkin-Huxley Model

**DOI:** 10.1155/2014/761907

**Published:** 2014-07-03

**Authors:** Yue Zhang, Kuanquan Wang, Yongfeng Yuan, Dong Sui, Henggui Zhang

**Affiliations:** ^1^Biocomputing Research Center, School of Computer Science and Technology, Harbin Institute of Technology, Harbin 150001, China; ^2^School of Physics & Astronomy, University of Manchester, Manchester, UK

## Abstract

Hodgkin-Huxley (HH) equation is the first cell computing model in the world and pioneered the use of model to study electrophysiological problems. The model consists of four differential equations which are based on the experimental data of ion channels. Maximal conductance is an important characteristic of different channels. In this study, mathematical method is used to investigate the importance of maximal sodium conductance ${\overset{-}{g}}_{\text{Na}}$ and maximal potassium conductance ${\overset{-}{g}}_{\text{K}}$. Applying stability theory, and taking ${\overset{-}{g}}_{\text{Na}}$ and ${\overset{-}{g}}_{\text{K}}$ as variables, we analyze the stability and bifurcations of the model. Bifurcations are found when the variables change, and bifurcation points and boundary are also calculated. There is only one bifurcation point when ${\overset{-}{g}}_{\text{Na}}$ is the variable, while there are two points when ${\overset{-}{g}}_{\text{K}}$ is variable. The (${\overset{-}{g}}_{\text{Na}}$,  ${\overset{-}{g}}_{\text{K}}$) plane is partitioned into two regions and the upper bifurcation boundary is similar to a line when both ${\overset{-}{g}}_{\text{Na}}$ and ${\overset{-}{g}}_{\text{K}}$ are variables. Numerical simulations illustrate the validity of the analysis. The results obtained could be helpful in studying relevant diseases caused by maximal conductance anomaly.

## 1. Introduction

Hodgkin-Huxley (HH) equation is created on the foundation of huge experimental data of sodium and potassium channels by Hodgkin and Huxley who are both excellent biology scientists and had long engaged in nerve conduction research. In about 1952, they took squid giant axon as experiment subject and continuously published four papers describing the electrical excitation of this kind of cell [1–4]. In their experiment, all the ion channels were divided into three types, sodium channel, potassium channel, and the others. Now we know there are many ion channels on the cell membrane, such as *I*_Na_, *I*_Kr_, *I*_Ks_, *I*_NaCa_, *I*_K1_, *I*_CaL_, *I*_Ca_, *I*_to_, *I*_NaK_, *I*_NaL_, and *I*_KATP_ [5–7]. However the discovery of sodium and potassium channels was marvelous at that time. Experimental data was obtained by voltage-clamp technology, while the patch-clamp technology is widely used at present. On this basis, a four-dimensional ordinary differential equation set, called HH model, was proposed, which was autonomous and contained intricate transcendental equations.

The work of Hodgkin and Huxley was recognized as excellent achievement and with significant contribution to the development of electrophysiology. It is the basis of the subsequent models of ion channels. Not only was the HH model consistent with the obtained experiment data accurately, but also it could precisely simulate the change of action potential. The model discovered the relationship of transmembrane potential and current and maximum conductance of ions. This made it possible to research the character of ion channel with mathematical methods. In 1960, Professor Nobel who pioneered the cardiac electrophysiology simulations applied HH model to myocardial cell and got the famous Purkinje fiber cell model [8], which was the first computing myocardial cell model. From then on, HH model was broadly applied to almost all kinds of cardiac cells such as atrial muscle cell model [9] and sinoatrial node cell model [10]. HH model laid the cornerstone of computing electrophysiology. Even today, a large part of electrophysiological models are created on the foundation of HH model. Verkerk's sinoatrial model [11], Butters's atrial model [12], O'Hara's ventricular model [13], and Li's Purkinje cell model [14], and so forth, all belong to HH model type.

Because of the importance of HH model, the stability has long attracted the researcher's attention. Hassard et al. were the earlier researchers caring about the bifurcation phenomenon of HH model. And they indicated that bifurcation would occur at the equilibrium points when the external current *i*_ext_ changed which was injected into the neuron from microelectrode [15]. Stable and unstable solutions of the model with regard to *i*_ext_ were analyzed by Rinzel and Miller, and the influence of temperature was also discussed [16]. Two stable steady states were found by Aihara and Matsumoto [17]; when the two states existed, the bifurcation structure was complex, which included a stable limit cycle, two unstable equilibrium points, and one asymptotically stable equilibrium point. Guckenheimer and Labouriau investigated the influence of *i*_ext_ and potassium ion potential *V*_K_ on the bifurcations of the model [18]. Bedrov et al. gave the relationship between the numbers of negative slope regions and presented some results about the possible bifurcation giving rise to maximal sodium conductance ${\overset{-}{g}}_{\text{Na}}$ and maximal potassium conductance ${\overset{-}{g}}_{\text{K}}$ [19, 20]. Fukai and his fellows examined the structure of the model's bifurcations produced by *i*_ext_ and one of the other parameters [21]. Taking leakage conductance *g*_*l*_ and sodium channel effective bias voltage ${\overset{-}{V}}_{m}$ as parameters, Terada et al. analyzed bifurcation in Hodgkin-Huxley model for muscles of frogs [22].

Wang et al. [23–26] did a lot of research on the stability of HH model. They studied the bifurcations caused by leakage conductance ${\overset{-}{g}}_{l}$ and sodium ions antielectromotive force when ELF external electric field was considered. The stability and bifurcation control were analyzed and controllers were designed. Bifurcation in HH model exposed to DC electric fields was investigated in detail.

Bifurcation means qualitative changes in the solution structure of a dynamic system when the parameters vary. From analyzing the bifurcation, we can get the effects of the parameters. Further, changing the corresponding parameters, we could make the solution into an ideal condition. Bifurcation is an important branch in mathematics and applied to much different field [27–29]. In recent years, it is also widely studied in electrophysiology. Indeed, there are many diseases having close relations with bifurcations, such as Parkinson's, epilepsy, and pathological heart rhythms [30].

In the past, for HH model, external current *i*_ext_ and leakage conductance ${\overset{-}{g}}_{l}$ have been most investigated, because they were easily measured. The sodium current is the contributor which leads to depolarization of the neuron while it is potassium current that plays the major role of repolarization. However, ${\overset{-}{g}}_{\text{Na}}$ and ${\overset{-}{g}}_{\text{K}}$ are seldom taken into consideration to analyze the stability of model, as the relevant data is not abundant. In this study, the effects of  ${\overset{-}{g}}_{\text{Na}}$ and ${\overset{-}{g}}_{\text{K}}$ on the stability and bifurcations of the model will be discussed, respectively, and collectively. And we will give the critical points of ${\overset{-}{g}}_{\text{Na}}$ and ${\overset{-}{g}}_{\text{K}}$ when they play the role separately, and the critical boundaries in ${\overset{-}{g}}_{\text{Na}}\text{-}{\overset{-}{g}}_{\text{K}}$ plane will be provided when together. Simulation results demonstrate the validity of the theoretic analysis.

The rest of the paper is organized as follows. The HH equations are introduced in detail in Section 2. In Section 3 we analyze the effects of ${\overset{-}{g}}_{\text{Na}}$ and ${\overset{-}{g}}_{\text{K}}$ on the model and calculate the bifurcation points (line). Finally, discussion and conclusion are presented in Section 4.

## 2. Hodgkin-Huxley Equations

**Figure 1:**
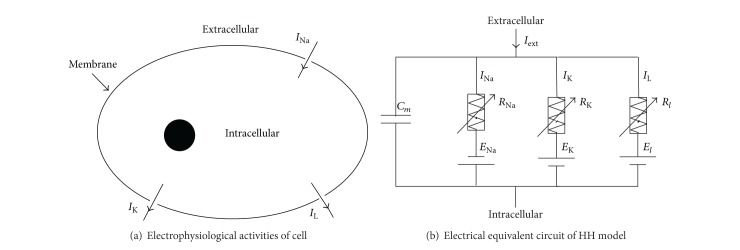
The electrophysiological process and equivalent circuit of neuron.

HH model was proposed on the foundation of ion channels. The electrophysiological activities of a cell are shown in Figure 1(a). The gray circle is membrane, which ensures orderly biochemical reaction. *I*_Na_, *I*_K_, and *I*_L_ are the ion currents corresponding to respective channels on the membrane. When an electrical stimulation makes the sodium channels open, a large number of Na^+^ flow inward, forming current *I*_Na_, resulting in the rise of transmembrane potential. The open of potassium channels makes a large outflux of K^+^, creating the current *I*_K_ and the reduction of potential. The model is comprised of four autonomous ordinary differential equations to describe the electrophysiological activities of cell shown in Figure 1(a). In the model, membrane is taken as a constant capacitance and the ion channels are seen as variable resistances.  Figure 1(b) shows the equivalent circuit in detail, in which *R*_Na_ = 1/*g*_Na_, *R*_K_ = 1/*g*_K_, and *R*_*l*_ = 1/*g*_*l*_. *R*_Na_ and *R*_K_ vary with time.

The equations were obtained according to electrical formulas and experimental data, which are shown as follows:

(1) dVdt=1CM[iext−g−Nam3h(V−VNa)−g−Kn4(V−VK)−g−l(V−Vl)],dmdt=αm(V)(1−m)−βm(V)m,dhdt=αh(V)(1−h)−βh(V)h,dndt=αn(V)(1−n)−βn(V)n,

where

(2) αm(V)=0.1(V−25.0)1−exp⁡⁡[−(V−25.0)/10],βm(V)=4.0exp⁡⁡(−V18.0),αh(V)=0.07exp⁡⁡(−V20.0),βh(V)=11+exp⁡⁡[−(V−30.0)/10],αn(V)=0.01(V−10.0)1−exp⁡⁡[−(V−10.0)/10],βn(V)=0.125exp⁡⁡(−V80.0).

In these equations, *V* is the transmembrane potential. 0 ≤ *m* ≤ 1 and 0 ≤ *h* ≤ 1 are the gating variables indicating activation and inactivation of sodium ion current, respectively. 0 ≤ *n* ≤ 1 is the gating variable showing activation of potassium ion current. ${\overset{-}{g}}_{\text{Na}}$, ${\overset{-}{g}}_{\text{K}}$, and ${\overset{-}{g}}_{l}$ represent the maximal conductance of corresponding currents. *C*_*m*_ = 1.0 *μ*F/cm^2^ is membrane capacitance. *i*_ext_ is the current injected into the neuron. In our paper, we suppose *i*_ext_ = 0 and ${\overset{-}{g}}_{\text{Na}}=120$ mS/cm^2^, ${\overset{-}{g}}_{\text{K}}=36$ mS/cm^2^, and ${\overset{-}{g}}_{l}=0.3$ mS/cm^2^, which are the ideal experimental data.

## 3. Stability Analysis of HH Model

Stability is one of a model's important properties. If the model is stable, it will reach a rest state at last. Otherwise, periodic phenomenon or chaos may appear. To analyze an ordinary differential system, equilibrium points are one of its most important aspects, which may be the final state of the system. Suppose (*V*_∗_, *m*_∗_, *h*_∗_, *n*_∗_) is the equilibrium points of the model. So it should make the right side of (1) equal to zero. That is,

(3) iext−g−Nam∗3h∗(V∗−VNa)−g−Kn∗4(V∗−VK) −g−l(V∗−Vl)=0,αm(V∗)(1−m∗)−βm(V∗)m∗=0,αh(V∗)(1−h∗)−βh(V∗)h∗=0,αn(V∗)(1−n∗)−βn(V∗)n∗=0.

Then the linearization of (1) around the equilibrium could be obtained as follows:

(4) dVdt=J11V+J12m+J13h+J14n,dmdt=J21V+J22m,dhdt=J31V+J33h,dndt=J41V+J44n,

where

(5) J11=−g−Nam∗3h∗+g−Kn∗4+g−lCM,J12=−3g−Nam∗2h∗(V∗−VNa)CM,J13=−g−Nam∗3(V∗−VNa)CM,J14=−4g−Kn∗3(V∗−VK)CM,J21=2m∗9exp⁡⁡(V∗/18.0) −{0.1(V∗−25.0)exp⁡⁡[−(V∗−25.0)/10][1−exp⁡⁡(−(V∗−25)/10)]2−0.11−exp⁡⁡(−(V∗−25)/10)}(1−m∗),J22=0.1(V∗−25.0)1−exp⁡⁡[−(V∗−25.0)/10]−4.0exp⁡⁡(−V∗18.0),J31=−7exp⁡⁡(V∗/20.0)2000(1−h∗) −exp⁡⁡(−(V∗−30.0)/10)h∗10[1+exp⁡⁡(−(V∗−30.0)/10)]2,J33=0.07exp⁡⁡(−V∗20.0)−11+exp⁡⁡(−(V∗−30.0)/10),J41=n∗exp⁡⁡(V∗/80.0)640 +{1100[1−exp⁡⁡(−(V∗−10.0)/10)]  −0.01exp⁡⁡[−(V∗−10.0)/10](V∗−10.0)10[1−exp⁡⁡(−(V∗−10.0)/10)]2}(1−n∗),J44=0.01(V∗−10.0)1−exp⁡⁡[−(V∗−10.0)/10]−0.125exp⁡⁡(−V∗80.0).

We can get the eigenmatrix of (4):

(6) $$\begin{array}{c} J=\left(\begin{array}{cccc} J_{11} & J_{12} & J_{13} & J_{14}\\ J_{21} & J_{22} & 0 & 0\\ J_{31} & 0 & J_{33} & 0\\ J_{41} & 0 & 0 & J_{44} \end{array}\right) \end{array}$$

and then the characteristic equation can be obtained:

(7) $$\begin{array}{c} \lambda^{4}+a\lambda^{3}+b\lambda^{2}+c\lambda +d=0, \end{array}$$

where

(8) a=−(J11+J22+J33+J44),b=J11(J22+J33+J44)+J22(J33+J44) +J33J44−J12J21−J13J31−J14J41,c=J12J21(J33+J44)+J13J31(J22+J44) +J14J41(J22+J33)−J11J22(J33+J44) −(J11+J22)J33J44,d=J11J22J33J44−J12J21J33J44−J13J22J31J44 −J14J22J33J41.

According to Routh-Hurwitz criterion, if *a* > 0, *ab* > *c*, *d* > 0,  *ab* *c* > *c* ^2^ + *a* ^2^ *d*, the real parts of all the roots are minus. Otherwise, all the real parts are not negative. In the following, we will analyze the stability of the model according to this criterion.

### 3.1. Effect of ${\overset{-}{g}}_{\text{Na}}$ on the Stability

**Figure 2:**
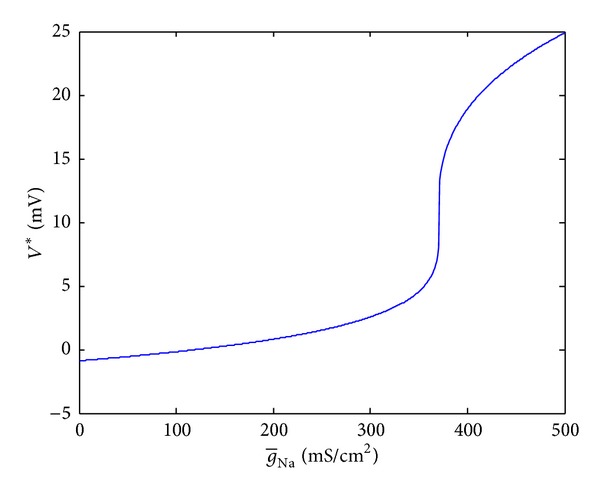
The relationship between ${\overset{-}{g}}_{\text{Na}}$ and V*.

In this section, we will investigate the influence of ${\overset{-}{g}}_{\text{Na}}$ on the equilibrium, stability, and bifurcations of the model. ${\overset{-}{g}}_{\text{Na}}$ is taken as variable, and the other parameters are all kept with desired values. Because the desired value of ${\overset{-}{g}}_{\text{Na}}$ is around 120 mS/cm^2^, ${\overset{-}{g}}_{\text{Na}}\in [0,500]$ is taken into consideration. When ${\overset{-}{g}}_{\text{Na}}$ changes, making the right side of (1) equal to zero, corresponding V* can be acquired. Taking Matlab as a tool, we could obtain the relationship between ${\overset{-}{g}}_{\text{Na}}$ and the equilibrium point V* shown in Figure 2.

From Figure 2, we can see that V* changes slowly when ${\overset{-}{g}}_{\text{Na}}\in [0,300]$ and increases rapidly when ${\overset{-}{g}}_{\text{Na}}\in [350,500]$. This means that equilibrium points are sensitive to ${\overset{-}{g}}_{\text{Na}}$ when ${\overset{-}{g}}_{\text{Na}}\in [350,500]$; a slight change of ${\overset{-}{g}}_{\text{Na}}$ may lead the model to a totally different state even though model is still stable.

Applying bifurcation theory and using the method of bisection, we can get one bifurcation point ${\overset{-}{g}}_{\text{Na}}^{*}=212.648720656$ when ${\overset{-}{g}}_{\text{Na}}$ changes.

Substituting ${\overset{-}{g}}_{\text{Na}}^{*}$ into the original equation, we get the equilibrium V*, and then substituting both ${\overset{-}{g}}_{\text{Na}}^{*}$ and V* into eigenmatrix of (4), we can gain the eigenvalues as follows:

(9) λ1=−4.9711711484,λ2=−0.1259717048,λ3=  1.9×10−16−0.3798402483i,λ4=1.9×10−16+0.3798402483i.

Here, we regard 1.9 × 10^−16^ as 0. With the help of computer, we can get that all the real parts of *λ*_*i*_ (*i* = 1, 2, 3, 4) are negative when ${\overset{-}{g}}_{\text{Na}}<{\overset{-}{g}}_{\text{Na}}^{*}$. And there exist positive real parts when ${\overset{-}{g}}_{\text{Na}}>{\overset{-}{g}}_{\text{Na}}^{*}$. According to the stability theory, the system is stable around equilibrium when ${\overset{-}{g}}_{\text{Na}}\in [0,{\overset{-}{g}}_{\text{Na}}^{*})$ and it is unstable when ${\overset{-}{g}}_{\text{Na}}\in ({\overset{-}{g}}_{\text{Na}}^{*},500]$. The model undergoes Hopf bifurcation at ${\overset{-}{g}}_{\text{Na}}={\overset{-}{g}}_{\text{Na}}^{*}$.

**Figure 3:**
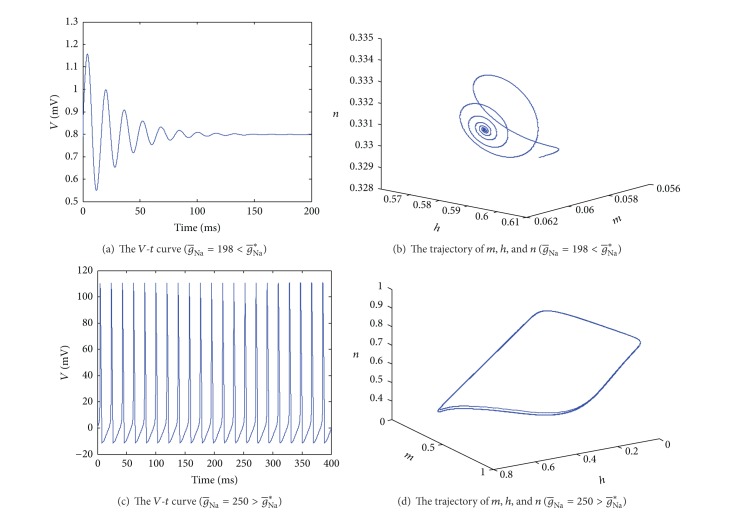
The response of *V* and *m*, *h*, and *n* to different ${\overset{-}{g}}_{\text{Na}}$.

Figure 3 shows the response of *V* and *m*, *h*, and *n* to different ${\overset{-}{g}}_{\text{Na}}$. As the analysis above, when ${\overset{-}{g}}_{\text{Na}}=198<{\overset{-}{g}}_{\text{Na}}^{*}$, the system is stable.  Figure 3(a) is the potential-time (*V*-*t*) curve, which shows that the action potential *V* becomes steady.  Figure 3(b) displays the trajectory of gating variables *m*, *h*, and *n* with time. We can see that the electrophysiological activity of cell reaches an equilibrium state at last.

Figures 3(c) and 3(d) demonstrate that the system is unstable when ${\overset{-}{g}}_{\text{Na}}=250>{\overset{-}{g}}_{\text{Na}}^{*}$.  Figure 3(c) is the *V*-*t* graph, from which we can see the potential changes periodically.  Figure 3(d) describes the trend of *m*, *h*, and *n*, whose trajectory is a circle finally. Both Figures 3(c) and 3(d) imply that the system is unstable and the electrophysiological activity of cell is in a periodical state at a certain frequency.

### 3.2. Effect of ${\overset{-}{g}}_{\text{K}}$ on the Model

**Figure 4:**
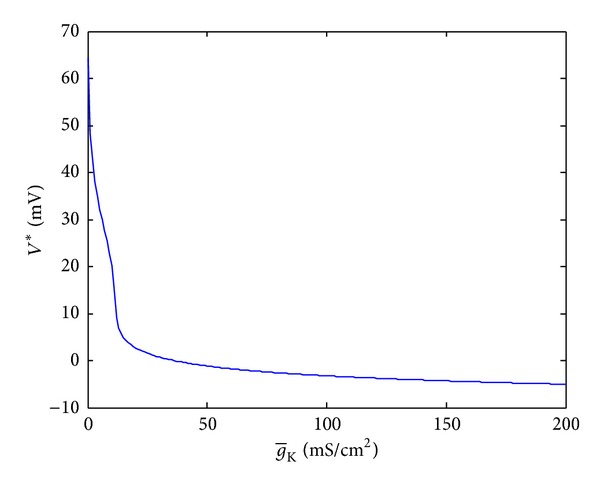
The relationship between ${\overset{-}{g}}_{\text{K}}$ and V*.

In this part, we choose ${\overset{-}{g}}_{\text{K}}$ as variable and keep the other parameters with ideal values. The same method with analysis of ${\overset{-}{g}}_{\text{Na}}$ is taken to analyze the effect of ${\overset{-}{g}}_{\text{K}}$ on the equilibrium, stability, and bifurcation of HH model. ${\overset{-}{g}}_{\text{K}}\in [0,200]$ is taken into consideration because the desired value of ${\overset{-}{g}}_{\text{K}}$ is 36.0. First, the relationship between ${\overset{-}{g}}_{\text{K}}$ and the equilibrium V* is obtained in Figure 4.

From Figure 4, we can see that V* varies rapidly when ${\overset{-}{g}}_{\text{K}}\in [0,20]$ and decreases slowly when ${\overset{-}{g}}_{\text{K}}\in [30,200]$. This means that equilibrium points are sensitive to ${\overset{-}{g}}_{\text{K}}$ when ${\overset{-}{g}}_{\text{K}}\in [0,20]$. A slight change of ${\overset{-}{g}}_{\text{K}}$ may make the final state of model change greatly.

Using the method of bisection to calculate the eigenvalues, we can find two bifurcation points ${{\overset{-}{g}}_{\text{K}}^{*}}_{1}^{}=3.843499029$ and ${{\overset{-}{g}}_{\text{K}}^{*}}_{2}^{}=19.762260771$ when ${\overset{-}{g}}_{\text{K}}$ varies. Substitute ${{\overset{-}{g}}_{\text{K}}^{*}}_{i}^{}$ (*i* = 1, 2) into (1), and obtain the corresponding equilibrium points V*. Both ${{\overset{-}{g}}_{\text{K}}^{*}}_{i}^{}$ and V* are substituted into (7), and then corresponding eigenvalues could be obtained as follows:

(10) $$\begin{array}{c} \lambda_{1}^{1}=-5.3218099843,\\ \lambda_{2}^{1}=-0.4223840650,\\ \lambda_{3}^{1}=3.2\times 10^{-16}-1.1305093754i,\\ \lambda_{4}^{1}=\;3.2\times 10^{-16}+1.1305093754i,\\ \lambda_{1}^{2}=-4.5370272278,\\ \lambda_{2}^{2}=-0.1319002182,\\ \lambda_{3}^{2}=4.2\times 10^{-16}-0.3436440068i,\\ \lambda_{4}^{2}=4.2\times 10^{-16}+0.3436440068i. \end{array}$$

**Figure 5:**
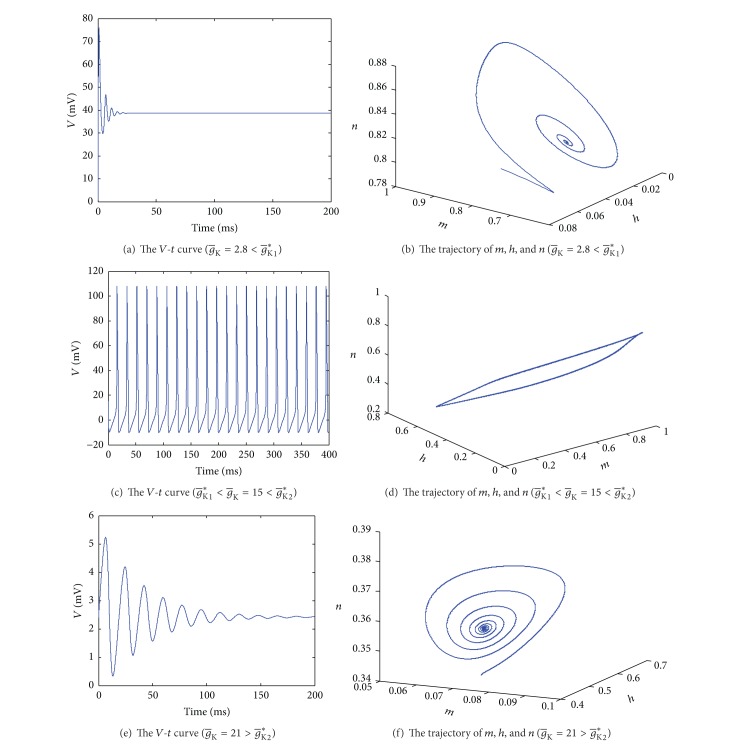
The response of *V* and *m*, *h*, and *n* to different ${\overset{-}{g}}_{\text{K}}$.

Here, 3.2 × 10^−16^ and 4.2 × 10^−16^ can be approximately regarded as 0. From computing, all the real parts of eigenvalues are negative when ${\overset{-}{g}}_{\text{K}}\in [0,{{\overset{-}{g}}_{\text{K}}^{*}}_{1}^{})\cup ({{\overset{-}{g}}_{\text{K}}^{*}}_{2}^{},200]$, and all of them are not negative when ${\overset{-}{g}}_{\text{K}}\in ({{\overset{-}{g}}_{\text{K}}^{*}}_{1}^{},{{\overset{-}{g}}_{\text{K}}^{*}}_{2}^{})$. According to the stability theory, the system is stable around equilibrium when ${\overset{-}{g}}_{\text{K}}\in [0,{{\overset{-}{g}}_{\text{K}}^{*}}_{1}^{})\cup ({{\overset{-}{g}}_{\text{K}}^{*}}_{2}^{},200]$ and it is unstable when ${\overset{-}{g}}_{\text{K}}\in ({{\overset{-}{g}}_{\text{K}}^{*}}_{1}^{},{{\overset{-}{g}}_{\text{K}}^{*}}_{2}^{})$. The model undergoes Hopf bifurcations at ${\overset{-}{g}}_{\text{K}}={{\overset{-}{g}}_{\text{K}}^{*}}_{i}^{}$ (*i* = 1, 2). The system is from locally stable state (${\overset{-}{g}}_{\text{K}}\in [0,{{\overset{-}{g}}_{\text{K}}^{*}}_{1}^{})$) to unstable state (${\overset{-}{g}}_{\text{K}}\in ({{\overset{-}{g}}_{\text{K}}^{*}}_{1}^{},{{\overset{-}{g}}_{\text{K}}^{*}}_{2}^{})$) and becomes stable (${\overset{-}{g}}_{\text{K}}\in ({{\overset{-}{g}}_{\text{K}}^{*}}_{2}^{},200]$) again. Responses of *V* and *m*, *h*, and *n* to different ${\overset{-}{g}}_{\text{K}}$ are shown in Figure 5.

Figure 5 shows the response of *V* and *m*, *h*, and *n* to different ${\overset{-}{g}}_{\text{K}}$. When ${\overset{-}{g}}_{\text{K}}=2.8<{{\overset{-}{g}}_{\text{K}}^{*}}_{1}^{}$, the system is stable.  Figure 5(a) shows the trend of potential with time, from which we can see that the potential reaches a fixed value.  Figure 5(b) is the trajectory of *m*, *h*, and *n* with time. All the gating variables also stay at fixed values (a steady point in Figure 5(b)) at last. These mean that the electrophysiological activity of cell reaches a steady state ultimately.

Figures 5(c) and 5(d) are *V*-*t* and *m*-*h*-*n* graphs, respectively, when ${{{\overset{-}{g}}_{\text{K}}^{*}}_{1}^{}<\overset{-}{g}}_{\text{K}}=15<{{\overset{-}{g}}_{\text{K}}^{*}}_{2}^{}$.  Figure 5(c) shows that the action potential changes in a certain period. And Figure 5(d) describes the trajectory of *m*, *h*, and *n* with time, from which we can find that the shape of the trajectory is a loop. Figures 5(c) and 5(d) imply all the gating variables and potential change periodically, which means that the electrophysiological activity of cell is in a periodical state.

Figures 5(e) and 5(f) are *V*-*t* and *m*-*h*-*n* curves, respectively, when ${\overset{-}{g}}_{\text{K}}=21>{{\overset{-}{g}}_{\text{K}}^{*}}_{2}^{}$. From Figure 5(e) we can see that the potential reaches the resting state at this occasion.  Figure 5(f) describes the trajectory of *m*, *h*, and *n*, which shows that the three variables stay at a fixed point at last. Both Figures 5(e) and 5(f) show that all the potential and gating variables no longer change with time, which implies the cell reaches the resting state finally.

### 3.3. Effect of ${\overset{-}{g}}_{\text{Na}}$ and ${\overset{-}{g}}_{\text{K}}$ on the Model

**Figure 6:**
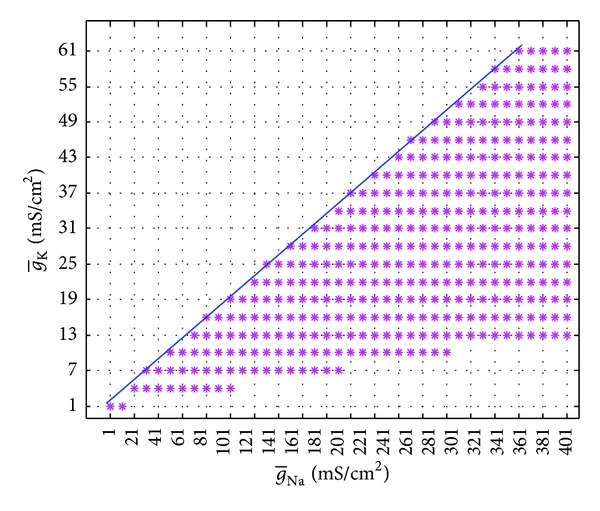
The ${\overset{-}{g}}_{\text{Na}}\text{-}{\overset{-}{g}}_{\text{K}}$ plane and the critical boundary.

Both ${\overset{-}{g}}_{\text{Na}}$ and ${\overset{-}{g}}_{\text{K}}$ are taken as variables in this part to study the stability and bifurcation of the model when ${\overset{-}{g}}_{\text{Na}}\in [0,400]$ and ${\overset{-}{g}}_{\text{K}}\in [0,60]$. Keeping the other parameters with desired values, using Matlab as a tool, we get the equilibrium points first when ${\overset{-}{g}}_{\text{Na}}$ and ${\overset{-}{g}}_{\text{K}}$ both vary. Then the points are substituted into eigenmatrix of (4) and the eigenvalues of the model can be calculated. At last, Figure 6 is gained, in which all the real parts of eigenmatrix are negative if ${\overset{-}{g}}_{\text{Na}}$ and ${\overset{-}{g}}_{\text{K}}$ belong to the pink region and positive real parts appear if ${\overset{-}{g}}_{\text{Na}}$ and ${\overset{-}{g}}_{\text{K}}$ are in white area.

From Figure 6, we can find that the upper boundary of the regions is similar to a line. With the least square method applied, the expression of the line can be gotten as ${\overset{-}{g}}_{\text{K}}=0.175\times {\overset{-}{g}}_{\text{Na}}-1.675$. However, the lower boundary is not regular. According to stability theory, we can easily know that the model is stable when ${\overset{-}{g}}_{\text{Na}}$ and ${\overset{-}{g}}_{\text{K}}$ are in pink region and unstable when ${\overset{-}{g}}_{\text{Na}}$ and ${\overset{-}{g}}_{\text{K}}$ are in white. This means that the electrophysiological activity can reach a steady state when ${\overset{-}{g}}_{\text{Na}}$ and ${\overset{-}{g}}_{\text{K}}$ are in pink region and it is periodic when ${\overset{-}{g}}_{\text{Na}}$ and ${\overset{-}{g}}_{\text{K}}$ are in white. The system undergoes bifurcations when ${\overset{-}{g}}_{\text{Na}}$ and ${\overset{-}{g}}_{\text{K}}$ are on the boundary.

## 4. Discussion and Conclusion

The effects of ${\overset{-}{g}}_{\text{Na}}$ and ${\overset{-}{g}}_{\text{K}}$ on the stability and bifurcation of HH model are analyzed in the paper. The critical values and boundary are obtained. When ${\overset{-}{g}}_{\text{Na}}$ increases to the critical value, the model will have bifurcation phenomenon, which means system will reach stable state when ${\overset{-}{g}}_{\text{Na}}$ is less than the critical value and the cell will have continuous action potential after stimulation when ${\overset{-}{g}}_{\text{Na}}$ is greater. However, there are two critical values about ${\overset{-}{g}}_{\text{K}}$. The system will be stable when ${\overset{-}{g}}_{\text{K}}$ is less than the smaller critical value and there are periodic solutions when ${\overset{-}{g}}_{\text{K}}$ is greater than the value and meanwhile is less than the larger one. The model will reach steady state again when ${\overset{-}{g}}_{\text{K}}$ is greater than the larger critical value. From analyzing ${\overset{-}{g}}_{\text{Na}}$ and ${\overset{-}{g}}_{\text{K}}$ collectively, we can get a critical line which divides the ${\overset{-}{g}}_{\text{Na}}\text{-}{\overset{-}{g}}_{\text{K}}$ plane into two parts. The system will be stable when $({\overset{-}{g}}_{\text{Na}}$, ${\overset{-}{g}}_{\text{K}})$ is in the upper half plane and model will have periodic solutions when $({\overset{-}{g}}_{\text{Na}}$, ${\overset{-}{g}}_{\text{K}})$ is in the lower half.

In our analysis, when ${\overset{-}{g}}_{\text{Na}}$ or ${\overset{-}{g}}_{\text{K}}$ are taken as the variable(s), all the other parameters are kept with desired values. However, almost all the biological systems are coupled. All the components influence one another and work together forming the overall functionality. Therefore, when the sodium (${\overset{-}{g}}_{\text{Na}}$) and/or potassium (${\overset{-}{g}}_{\text{K}}$) channels vary, do the other parameters remain unchanged? Is it reasonable to keep the other parameters still with desired values? We could not ensure that it must be reasonable. Nevertheless, some evidences may explain a certain rationality of the method. For example, tetrodotoxin (TTX) selectively binds to the outer vestibule voltage-gated sodium channels, preventing channels from opening [31]. Ivabradine is a sinus node *I*_*f*_ channel inhibitor, which is selective for the *I*_*f*_ current but does not affect other cardiac ionic currents [32]. Acacetin could suppress the ultrarapid delayed rectifier K^+^ current and the transient outward K^+^ current and block the acetylcholine-activated K^+^ current; however, it has no effect on Na^+^ current, L-type Ca^2+^ current, or even inward-rectifier K^+^ current [33]. All of these demonstrate that to an extent when one channel changes, the others may not be affected. That is, when the parameter describing a channel varies, it is reasonable to keep parameters describing the other channels unchanged.

Stable states indicate that the electrophysiological activity of cell will get to corresponding resting state at last, while periodic phenomenon looks like response of pathological cell's action potentials caused by cardiac arrhythmias [34]. In other words, ${\overset{-}{g}}_{\text{Na}}$ and ${\overset{-}{g}}_{\text{K}}$ may be the causes of the similar diseases to cardiac arrhythmias. So given appropriate medicine to change ${\overset{-}{g}}_{\text{Na}}$ or ${\overset{-}{g}}_{\text{K}}$ to reasonable intervals, the corresponding diseases could be abolished or the discomfort can be ameliorated. After all, our research could be a reference to treat relevant diseases. Some diseases led to by abnormal ion channels may be eased by medicine to adjust the conductance into corresponding intervals.
